# Combination Therapeutic Delivery Systems

**DOI:** 10.3390/pharmaceutics18070865

**Published:** 2026-07-16

**Authors:** Qingxin Mu

**Affiliations:** Department of Pharmaceutics, School of Pharmacy, University of Washington, Seattle, WA 98195, USA; qmu@uw.edu

Combination therapy has evolved rapidly over the past few decades into a powerful strategy to address complex health challenges such as cancer [1,2,3,4]. By targeting multiple mechanisms simultaneously, these approaches aim to overcome the limitations of single-agent treatments, including suboptimal efficacy, drug resistance, and dose-limiting toxicity [5,6]. Advanced delivery systems, which can be engineered to coordinate the in vivo dispositions of therapeutic agents, may further enhance their effectiveness [7,8,9,10,11]. This Special Issue showcases innovative research and reviews on combination therapeutic delivery, with a particular emphasis on nanoparticle advancements in co-formulating multiple agents, optimizing drug delivery profiles, and improving treatment outcomes.

In one study, Huang et al. investigated the co-delivery of paclitaxel (PTX), a hydrophobic chemotherapeutic, and polyinosinic–polycytidylic acid (Poly IC), a hydrophilic immunologic agent, using chitosan–PEG (CP) nanoparticles [12]. Their approach demonstrated the ability to co-formulate physicochemically incompatible molecules. The CP-PTX-Poly IC nanoparticles showed notable efficacy to breast, liver, and pancreatic cancer by enhancing cancer cell uptake and cellular potency. The nanoparticles also activated dendritic cells, as evidenced by increased CD80+ and CD86+ populations, while exhibiting low toxicity to the immune cells. These results underscore the potential of dual-modal delivery systems to simultaneously tackle cancer cells and activate the anti-cancer immune response. In another study, Adekiya et al. co-formulated docetaxel, a widely used chemotherapy drug, with brusatol, a multitarget inhibitor designed to overcome chemoresistance [13]. Using polymeric nanoparticles and a one-factor-at-a-time method, the researchers optimized the formulation to achieve a mean size of ~169 nm and sustained drug release over 24 h. This combination demonstrated synergistic effects in LNCaP and PC-3 prostate cancer cells yet differential effects on their cell cycle, caspase activity and survivin expression, offering a promising strategy to combat resistance and improve the therapeutic efficacy in heterogeneous prostate cancer. To improve the chemoimmunotherapy of osimertinib-resistant lung cancer, Nathani et al. examined the combined role of IL-15-stimulated natural killer (NK) cell-derived extracellular vesicles (EVs) and carboplatin [14]. Characterized by nanoparticle shape and size, and proteins involved in NK cell-mediated cytotoxicity and T cell activation, NK-EVs reduced the IC50 of carboplatin in resistant H1975R lung cancer cells. In vivo, this combination significantly reduced tumor volumes in a xenograft model compared to single treatments, demonstrating that NK-EVs can enhance the efficacy of traditional chemotherapy while leveraging immunologic pathways to target resistant cancers. A fourth study by Genc et al. highlights the use of magnetic Fe_3_O_4_ nanoparticles in combination with 5-fluorouracil (5-FU) for the treatment of colon cancer [15]. This combination enhanced oxidative stress within cancer cells, resulting in higher cytotoxicity and reduced cell viability at lower drug concentrations compared to 5-FU alone. The findings suggest that incorporating nanomaterials with traditional chemotherapeutics may enhance efficacy, reduce dosage requirements, and minimize toxicity.

In addition to original research, this Special Issue includes insightful reviews. One review by Liu et al. discussed the integration of photodynamic therapy (PDT) and photothermal therapy (PTT) with nanomedicine [16]. PDT offers minimal invasiveness and high selectivity but is limited by tumor hypoxia, which has led to its combination with PTT to enhance treatment outcomes. This review discussed the benefits of combining PDT and PTT with chemotherapy using smart nanomedicines that respond to tumor microenvironment stimuli, highlighting design innovations, challenges, and future potential in improving tumor treatment. In another review, Xu et al. focused on pain treatment and relief applications of nanomaterial-based delivery systems. By co-formulating multiple analgesics within a single nanoformulation, the controlled analgesic release approach can achieve sustained pain relief with prolonged analgesic effects and reduced administration frequency by harnessing the synergy and co-action of multiple targets, potentially transforming pain management [17]. A review on Pluronic F-68 and F-127-based nanomedicine by Khaliq et al. discusses their versatility in advancing combination therapy. These FDA-approved amphiphilic carriers not only improve the solubility of hydrophobic drugs but also enable co-administration of multiple agents for synergistic therapeutic effects [18]. Finally, He et al. reviewed advancements in rational modified nanocarriers for cytosolic protein delivery. Protein therapies offer high potency, specificity, and low toxicity but face challenges such as poor membrane penetration and inefficient intracellular delivery. Their review discusses advances in nanocarriers, such as liposomes and polymeric nanoparticles, to address these limitations and highlights strategies for enhancing cytosolic protein delivery, offering insights into future innovations for protein-based therapies including combination therapy [19].

In summary, this Special Issue has compiled compelling research findings and comprehensive reviews on combination therapeutic delivery systems, highlighting the value of co-formulating agents, particularly through nanoparticle-based approaches, in advancing combination therapy. These studies underscore the transformative potential of advanced delivery approaches in expanding treatment options and overcoming barriers to effective disease management. However, challenges remain, including formulation complexity, precise targeting, and mechanistic understanding of the delivery systems. As the field continues to evolve, further investigations into clinically translatable and mechanistically well-characterized co-delivery strategies are essential to bring these promising therapies closer to benefiting human health.
